# Effects of Aging on Levo-Dihydroxyphenylalanine- Induced Dyskinesia in a Rat Model of Parkinson’s Disease

**DOI:** 10.3389/fnagi.2021.650350

**Published:** 2021-05-13

**Authors:** Haruo Nishijima, Tamaki Kimura, Fumiaki Mori, Koichi Wakabayashi, Iku Kinoshita, Takashi Nakamura, Tomoya Kon, Chieko Suzuki, Masahiko Tomiyama

**Affiliations:** ^1^Department of Neurology, Institute of Brain Science, Hirosaki University Graduate School of Medicine, Hirosaki, Japan; ^2^Department of Neurology, National Hospital Organization, Aomori Hospital, Aomori, Japan; ^3^Department of Neuropathology, Institute of Brain Science, Hirosaki University Graduate School of Medicine, Hirosaki, Japan

**Keywords:** 6-hydroxydopamine (6-OHDA), abnormal involuntary movement, dynorphin, medial globus pallidus, young-onset Parkinson’s disease

## Abstract

**Background:**

It remains unclear why patients with young-onset Parkinson’s disease more often develop levo-dihydroxyphenylalanine (L-dopa)-induced dyskinesia (LID) and have a more severe form than patients with old-onset Parkinson’s disease. Previous studies using animal models have failed to show young-onset Parkinson’s disease enhances LID.

**Objectives:**

To evaluate the association of age at dopaminergic denervation (onset age) and initiation of L-dopa treatment (treatment age) with LID development in model rats.

**Methods:**

We established rat models of young- and old-lesioned Parkinson’s disease (6-hydroxydopamine lesions at 10 and 88 weeks of age, respectively). Dopaminergic denervation was confirmed by the rotational behavior test using apomorphine. Rats in the young-lesioned group were allocated to either L-dopa treatment at a young or old age, or saline treatment. Rats in the old-lesioned group were allocated to either L-dopa treatment or saline group. We evaluated L-dopa-induced abnormal involuntary movements during the 14-day treatment period. We also examined preprodynorphin mRNA expression in the striatum (a neurochemical hallmark of LID) and the volume of the medial globus pallidus (a pathological hallmark of LID).

**Results:**

LID-like behavior was enhanced in L-dopa-treated young-lesioned rats compared with L-dopa-treated old-lesioned rats. Preprodynorphin mRNA expression was higher in L-dopa-treated young-lesioned rats than in in L-dopa-treated old-lesioned rats. The volume of the medial globus pallidus was greater in L-dopa-treated young-lesioned rats than in L-dopa-treated old-lesioned rats. Treatment age did not affect LID-like behavior or the degree of medial globus pallidus hypertrophy in the young-lesioned model.

**Conclusion:**

Both dopaminergic denervation and L-dopa initiation at a young age contributed to the development of LID; however, the former may be a more important factor.

## Introduction

Parkinson’s disease is a neurodegenerative disorder characterized by motor symptoms such as tremor, akinesia, hypokinesia, rigidity, and postural disturbance (Nussbaum and Ellis, 2003). The most effective drug in the treatment of PD is L-dopa, a dopamine precursor (Agid et al., 1999; Nutt, 2008; Smith et al., 2012). However, chronic repetitive L-dopa treatment induces uncontrollable AIMs known as LID (Quinn, 1995; Rascol, 2000; Tran et al., 2018). LID is considered to be associated with L-dopa-induced maladaptive neuronal plasticity caused by intermittent unphysiological stimulation of dopamine receptors in the dopamine-denervated striatum (Bastide et al., 2015; Borgkvist et al., 2018). The threshold of L-dopa dose for LID expression is reduced immediately after dopaminergic denervation and after repetitive L-dopa treatment (Cenci and Lundblad, 2006). Specifically, LID development is determined by two factors: dopaminergic denervation (lesion-induced plasticity) and priming (L-dopa-induced plasticity).

In the 2000s, an L-dopa-sparing strategy was recommended to prevent the onset of motor complications, especially in patients with early-onset PD (Rascol et al., 2000; Holloway et al., 2004; Hauser et al., 2007). Recent studies indicate that early L-dopa treatment does not necessarily increase LID expression (Katzenschlager et al., 2008; PD Med Collaborative Group, 2014). A clinical study on a Ghanaian and Italian PD cohort reported no association between LID expression and duration of L-dopa treatment; instead, the predictors of motor complications were longer disease duration and a higher daily dose of L-dopa (Cilia et al., 2014).

Clinically, young-onset PD involves earlier, more frequent, and severe LID than old-onset PD (Schrag and Quinn, 2000; Kumar et al., 2005; Ku and Glass, 2010; Olanow et al., 2013). However, the mechanism underlying this phenomenon remains unclear. A higher incidence of LID in patients with young-onset PD may result due to dopaminergic denervation and/or L-dopa treatment at a young age. Although it is not clear which factor is more important for dyskinesia development, the Ghanaian study suggests that denervation age is a more important factor than treatment age.

Young adult rats with 6-OHDA-induced lesions have been used to study LID; however, few studies have used older rats. Only two studies have investigated the association between aging and LID development using rodent old-lesioned PD models (Bez et al., 2016; Lanza et al., 2019), neither of which indicated that the young-lesioned PD model was more likely to present with LID.

This study aimed to examine whether the young-lesioned PD model rat is more susceptible to LID than the old-lesioned model rat. In particular, we evaluated the association of age at dopaminergic denervation (onset age) and initiation of L-dopa treatment (treatment age) with the development of LID. Further, we examined the striatal preprodynorphin mRNA expression (a neurochemical LID hallmark) (Cenci et al., 1998) and the volume of the MGP (a pathological LID hallmark) (Tomiyama et al., 2004; Nishijima et al., 2020) in model rats.

## Materials and Methods

### Experimental Animals

This study used young (10 weeks old, weight: 260–280 g) and old (88 weeks old, weight: 550–640 g) male Wistar rats. All experiments were performed according to the Guidelines for Animal Experimentation issued by the Hirosaki University School of Medicine and the Guide for the Care and Use of Laboratory Animals (National Institutes of Health [NIH], United States). Animals were housed in a temperature-controlled room (approximately 25°C) with 12-h light-dark cycles and *ad libitum* access to food and water. All efforts were made to minimize the number of animals and their suffering.

### 6-Hydroxydopamine Lesions

To establish rat PD models, lesions in the dopaminergic system were generated on the right brain hemisphere in the YL and OL groups through 6-OHDA injection into the right medial forebrain bundle, as previously described (Nishijima et al., 2020). Briefly, following anesthesia by intraperitoneal injection of pentobarbital (50 mg/kg body weight), the rat head was fixed on a stereotactic apparatus (David Kopf, United States) with the incisor bar set 3.3 mm below the horizontal. Thirty minutes before 6-OHDA injection, the rat was intraperitoneally injected with desipramine (25 mg/kg) to prevent noradrenergic neuron denervation. Next, 6-OHDA was injected using a stainless-steel needle (0.4 mm outer diameter) inserted through a small burr hole in the right side of the skull. The needle tip was placed 4.5 mm posterior to the bregma, 1.2 mm lateral to the sagittal suture, and 9 mm ventral to the skull surface according to the atlas of Paxinos and Watson (1998). Subsequently, 6-OHDA (8 μg/4 μL in saline with 0.01% ascorbic acid) was injected over 4 min, with the needle being left in place for another 4 min to prevent backflow leakage.

To evaluate the extent of 6-OHDA lesioning, rats underwent rotational behavior testing at two post-operative weeks. Apomorphine (0.05 mg/kg) was subcutaneously administered, and the rats were placed in a stainless-steel bowl after 10 min. After a 5-min accommodation period, the number of turns-to-the left (the side contralateral to the lesion) made by the rat was counted for 5 min. More than 20 contralateral turns indicated severe dopaminergic denervation; rats meeting this criterion are shown to have lost >99% of dopamine in the striatum (Tanaka et al., 1999). We performed 6-OHDA lesioning in 24 young rats and 12 old rats. Eighteen of 24 young-lesioned rats (75%) passed the apomorphine test criteria and were included in the present study. Six rats that did not pass the test were excluded from this study. All 12 old-lesioned rats passed the apomorphine test. Altogether, 30 rats were successfully lesioned with 6-OHDA (18 young-lesioned and 12 old-lesioned).

### Drug Treatment

**FIGURE 1:**
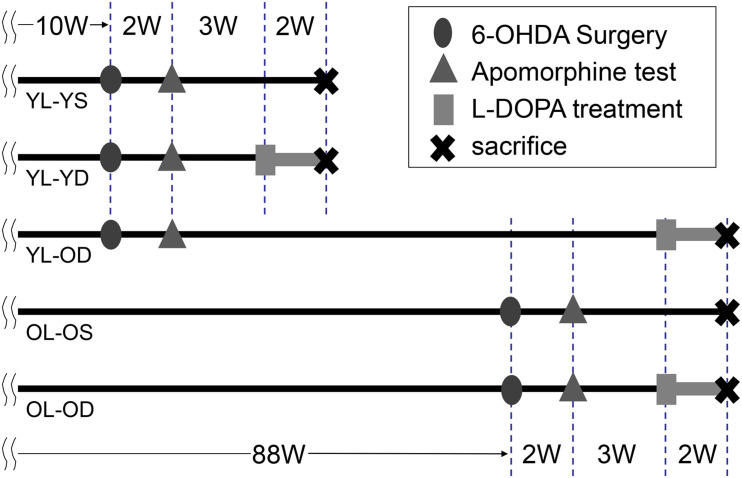
Time chart and experimental design of the study. We performed 6-OHDA surgery to develop hemi-parkinsonian rat models at 10 or 88 weeks of age. Dopaminergic denervation was confirmed by apomorphine test. The animals were then divided into five experimental groups. Rats received either L-dopa or saline daily and were sacrificed after the last treatment. 6-OHDA, 6-hydroxydopamine; L-dopa, levo-dihydroxyphenylalanine; OL-OD, old-lesioned old-L-dopa group; OL-OS, old-lesioned old-saline group; YL-OD, young-lesioned old-L-dopa group; YL-YD, young-lesioned young-L-dopa group; YL-YS, young-lesioned young-saline group, W, week.

We randomly allocated 18 young-lesioned PD model rats to daily receive two intraperitoneal injections (morning and evening) of L-dopa (10 mg/kg) with benserazide (5 mg/kg) 3 weeks after the apomorphine challenge (from 15 weeks of age) (young-lesioned young-L-dopa, YL-YD group, *n* = 6), L-dopa (10 mg/kg) with benserazide (5 mg/kg) 81 weeks after the apomorphine challenge (from 93 weeks of age) (young-lesioned old-L-dopa, YL-OD group, *n* = 6), and saline 3 weeks after apomorphine challenge (young-lesioned young-saline, YL-YS group, *n* = 6) for 2 weeks (Figure 1).

Furthermore, 12 old-lesioned PD model rats were randomly allocated to receive either intraperitoneal injections of L-dopa (10 mg/kg) with benserazide (5 mg/kg) (from 93 weeks of age) (old-lesioned old-L-dopa, OL-OD group, *n* = 6) or saline injections (old-lesioned old-saline, OL-OS group, *n* = 6) 3 weeks after the apomorphine challenge twice daily for 2 weeks.

Figure 1 presents the timeline and experimental design for these five groups. Differences between YL-YD versus YL-OD, YL-OD versus OL-OD, or YL-YD versus OL-OD reflected the age-related L-dopa treatment-induced plasticity, age-related denervation-induced plasticity, or both, respectively.

### Behavioral Analyses

L-Dopa-induced AIMs were recorded in the YL-YD, YL-OD, and OL-OD groups on days 1, 7, and 14. Saline-treated rats did not show AIMs. To evaluate AIMs, each rat was observed for a monitoring period of 1-min at 20-min intervals for 3 h after L-dopa injection. AIM scores were measured as described by Cenci et al. (1998). Abnormal repetitive movements that affected the body on the side contralateral to the lesion were classified as axial dystonia (contralateral twisted posturing of the neck and upper body), forelimb dyskinesia (repetitive contralateral forelimb jerks), or orolingual dyskinesia (jaw movements and contralateral tongue protrusion). Each rat was scored on a 4-point severity scale by two independent examiners blinded to animal treatment conditions. The sum of the axial, forelimb, and orolingual scores (ALO AIM score) was used as the LID index (Supplementary Video 1, mild dyskinesia; Supplementary Video 2, severe dyskinesia).

Further, we measured the total number of rotations to the contralateral side of the 6-OHDA lesion during 120 min after L-dopa injection on days 1, 7, and 14. This abnormal locomotory behavior can be induced by L-dopa and long-acting dopamine agonists (Lundblad et al., 2002; Ravenscroft et al., 2004), and thus, it is not a specific measure of L-dopa-induced motor complications (Henry et al., 1998; Lundblad et al., 2002; Cenci and Crossman, 2018). Instead, we used this number as an index of the priming effects of repetitive L-dopa treatment on PD rats (Supplementary Video 3, mild to moderate rotational behavior; Supplementary Video 4, severe rotational behavior).

### *In situ* Hybridization Histochemistry

Striatal dynorphin expression is associated with L-dopa-induced dyskinetic symptoms in PD rat models (Cenci et al., 1998). We examined preprodynorphin mRNA expression in the striatum of rats in each group using *in situ* hybridization histochemistry as previously described (Tomiyama et al., 1996, 2005).

Rats were sacrificed by decapitation 12 h after the last L-dopa injection on treatment day 14. Their brains were immediately removed, frozen on powdered dry ice, and stored at −30°C. Coronal striatal sections of 14-μm thickness were obtained on a cryostat (Microm HM500 OM, Thermo Fisher Scientific, Walldorf, Germany). Sections were thaw-mounted onto APS-coated glass slides (Matsunami, Osaka, Japan) and used for *in situ* hybridization histochemical analysis to detect rat mRNA coding for preprodynorphin. The specific oligonucleotide probe used for *in situ* hybridization for rat preprodynorphin mRNA consisted of 867–914 bases (Civelli et al., 1985). It was synthesized (380B Applied Biosystems DNA synthesizer, CA, United States) and purified on 20% polyacrylamide/8 M urea preparative sequencing gels. The probe was labeled using [33P]adATP (2000 Ci/mmol; DuPont-NEN, DE, United States) and terminal deoxynucleotidyltransferase (Boehringer Mannheim, Baden-Wurttemberg, Germany). The labeled probe was purified using the QIAquick Nucleotide Removal Kit (Qiagen GmbH, Hilden, Germany). The hybridized sections were exposed to Kodak BioMAX MR films at −80°C for 2–10 days with a Kodak MS screen to increase the hybridization signal intensity. The hybridization signal specificity was assessed as follows. First, for a given oligonucleotide probe, the presence of a 50-fold excess of unlabeled versus labeled probe resulted in signal abolition. Second, hybridized sections were washed at increasing temperatures and only hybridization signals present and absent in sections washed at 70°C and 80°C, respectively, were counted (because no decrease in signal was indicative of background noise). The levels of labeled preprodynorphin mRNA in the dorsal and ventral striatum were quantified by computerized densitometry. This was performed by scanning the labeled autoradiographic films (Epson GT-9500 scanner, Epson, Nagano, Japan) into a PowerMac G4 computer system (Apple Computer, CA, United States) and the images were analyzed using NIH Image 1.61 Software (NIH, MD, United States).

### Measurement of the MGP Volume

In the present study, to simplify the discussion of similarities between primates and rodents, we use the same terminology for rodents that is used in primates for the globus pallidus subdivisions. The structure known as the entopeduncular nucleus in rodents is termed as the MGP, i.e., the internal segment of the globus pallidus as per widely accepted homology (Paxinos and Watson, 1998).

MGP volume was examined as previously described (Gundersen et al., 1988; Tomiyama et al., 2004; Nishijima et al., 2020). Briefly, rats were sacrificed by decapitation 12 h after the last L-dopa treatment. Their brains were immediately removed, frozen on powdered dry ice, and stored at −80°C. Brains were sectioned on a cryostat (Microm) into 14-μm-thick slices in the coronal plane that passed through the MGP and thaw-mounted onto APS-coated glass slides (Matsunami). Every fourth section (10 sections in total) was stained using the Kluver-Barrera method for examination using the Olympus model SHZ-ILLB microscope (Olympus, Tokyo, Japan). MacSCOPE computer software (Mitani Co. Ltd., Tokyo, Japan) with a PowerMac G4 computer system (Apple) was used to digitize the images and measure the total area and gray matter area of the MGP in each section. MGP volume was calculated using the MGP area visible in each of the 10 sections and the distance between sections. The ratio of the MGP volume (total and gray matter) on the lesioned side to that on the intact side was calculated and compared among experimental groups.

### Tyrosine Hydroxylase Immunohistochemistry

To confirm the extent of dopaminergic denervation in 6-OHDA-lesioned rats (YL-YD, YL-OD, and OL-OD groups), brain sections including the striatum were immunostained with monoclonal antibodies against tyrosine hydroxylase (TH16; Sigma, MO, United States; 1:3000) based on the ABC method using the Vectastain ABC kit (Vector, CA, United States).

### Statistical Analysis

Statistical analyses were performed using BellCurve for Excel version 3.20 (Social Survey Research Information Co., Ltd., Tokyo, Japan). All data are expressed as mean ± standard error of the mean. Between-group differences in the total number of rotations after L-dopa injection on days 1, 7, and 14 were evaluated using the two-way repeated measures ANOVA followed by Bonferroni *post hoc* test. Furthermore, between-group differences in the AIM scores on days 1, 7, and 14, as well as between-group differences in preprodynorphin mRNA expression in the dorsal and ventral striatum (expressed as optical density units) were evaluated using the two-way repeated measures ANOVA followed by Bonferroni *post hoc* test. We examined the association between ALO AIM score and preprodynorphin mRNA expression at the individual level using scatterplots for all rats, and Spearman’s rank correlation coefficient was used to assess the correlation. Between-group differences in the volume ratios of MGP (total and gray matter) were assessed using the one-way ANOVA followed by *post hoc* comparisons with Tukey–Kramer test. *P* < 0.05 was considered statistically significant.

## Results

### Behavioral Analyses

#### Rotation Behavior

**FIGURE 2:**
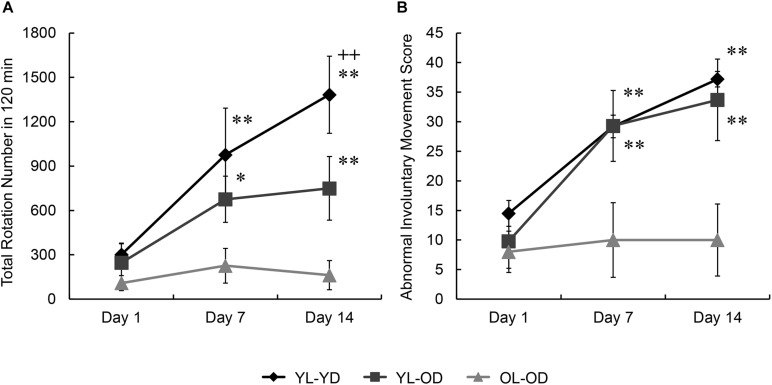
Rotational behavior and abnormal involuntary movement induced by repetitive L-dopa treatment in three groups of Parkinson’s disease rat model. **(A)** No between-group differences in the total number of rotations on day 1. On days 7 and 14, the YL-YD and YL-OD groups showed a higher number of rotations than the OL-OD group. On day 14, the YL-YD group showed a higher number of rotations than the YL-OD group. **(B)** No between-group differences in ALO AIM scores on day 1. On days 7 and 14, the YL-YD and YL-OD groups showed higher scores than the OL-OD group. Scores of the YL-YD and YL-OD groups were not different. *p < 0.05; **p < 0.01 compared with the OL-OD group on the treatment day; ^+ +^ *p* < 0.01 compared with the YL-OD group on the treatment day. Error bars represent the standard error of the mean. ALO AIM score, sum score of axial, forelimb, and orolingual scores; L-dopa, levo-dihydroxyphenylalanine; OL-OD, old-lesioned old-L-dopa group; YL-OD, young-lesioned old-L-dopa group; YL-YD, young-lesioned young-L-dopa group.

Most 6-OHDA-lesioned hemi-parkinsonian rats injected with L-dopa displayed rotational behavior to the left (contralateral to the lesion). We found significant effects of groups and treatment days on rotational behavior (groups, *F*[2,15] = 5.88, *p* = 0.013; days, *F*[2,30] = 16.65, *p* < 0.001; groups^∗^days, *F*[4,30] = 4.62, *p* = 0.005 by two-way repeated measures ANOVA). There was no between-group difference in the mean of the total number of rotations during 120 min after L-dopa injection on treatment day 1 (*p* = 1.000 for YL-YD versus YL-OD group, *p* = 0.596 for YL-YD versus OL-OD group, *p* = 1.000 for YL-OD versus OL-OD group by Bonferroni *post hoc* test) (Figure 2A), suggesting the degrees of dopaminergic denervation were not different between the three L-dopa-treated groups. On treatment days 7 and 14, the YL-YD and YL-OD groups showed a significant increase in the number of rotations compared with those on day 1 and those of the OL-OD group on each treatment day (*p* < 0.001 for day 1 versus day 7 and day 1 versus day 14 in YL-YD group, *p* < 0.001 for day 1 versus day 7 and day 1 versus day 14 in YL-OD group, *p* < 0.001 for YL-YD versus OL-OD group on days 7 and 14, *p* = 0.014 for YL-OD versus OL-OD group on day 7, *p* = 0.001 for YL-OD versus OL-OD group on day 14) (Figure 2A). Additionally, on treatment day 14, the number of rotations in the YL-YD group was significantly higher than that of the YL-OD group (*p* < 0.001) (Figure 2A). Thus, both dopaminergic denervation and L-dopa treatment at a young age contributed to the enhanced rotational behavior induced by chronic repetitive L-dopa injections.

#### ALO AIM Score

We found significant effects of groups and treatment days on ALO AIM scores (groups, *F*[2,15] = 4.81, *p* = 0.024; days, *F*[2,30] = 36.30, *p* < 0.001; groups^∗^days, *F*[4,30] = 7.10, *p* < 0.001 by two-way repeated measures ANOVA). There was no between-group difference in the ALO AIM score (an LID index) during the first day of L-dopa treatment (*p* = 0.729 for YL-YD versus YL-OD group, *p* = 0.327 for YL-YD versus OL-OD group, *p* = 1.000 for YL-OD versus OL-OD group by Bonferroni *post hoc* test) (Figure 2B). On treatment days 7 and 14, the YL-YD and YL-OD groups showed significantly increased ALO AIM scores compared with those on day 1 of each group and those of the OL-OD group on each treatment day (*p* < 0.001 for day 1 versus day 7 and day 1 versus day 14 in YL-YD group, *p* < 0.001 for day 1 versus day 7 and day 1 versus day 14 in YL-OD group, *p* < 0.001 for YL-YD versus OL-OD group on days 7 and 14, *p* < 0.001 for YL-OD versus OL-OD group on days 7 and 14) (Figure 2B). There were no significant differences between the YL-YD and YL-OD groups during 14 days of treatment (*p* = 1.000 on days 7 and 14) (Figure 2B). These results suggest that dopaminergic denervation age, but not the treatment age, contributes to the severity of LID-like involuntary movement in PD model rats.

#### Preprodynorphin mRNA Expression

**FIGURE 3:**
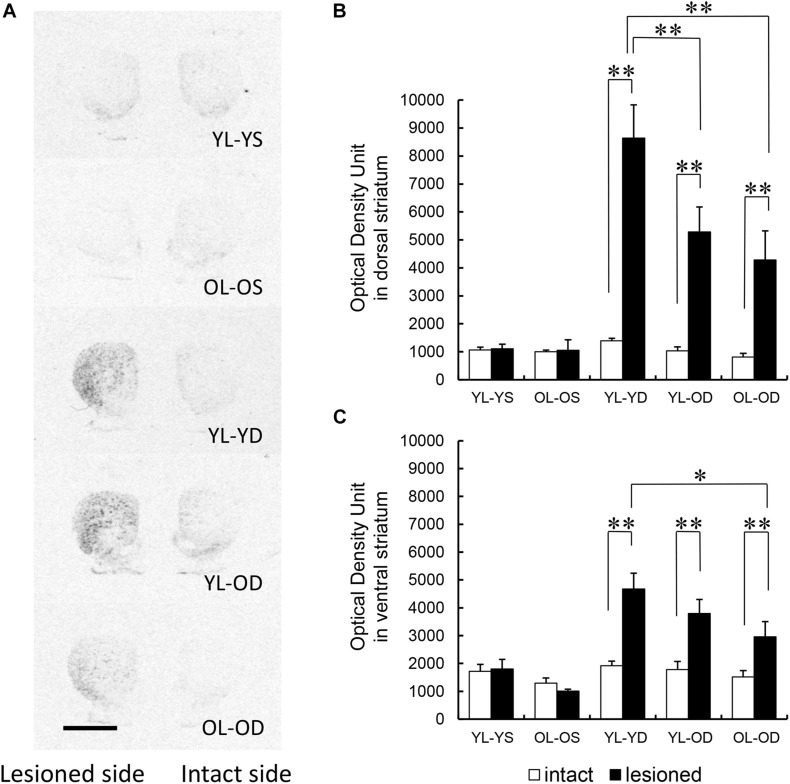
Regional distribution of striatal preprodynorphin mRNA expression in five experimental groups. **(A)** Representative autoradiograph images of the rat striatum from the five experimental groups. Dark areas correspond to regions rich in hybridization signals. Scale bar, 3 mm. **(B)** Preprodynorphin mRNA expression in the dorsal striatum of lesioned side was increased in the YL-YD, YL-OD, and OL-OD groups compared with that on the intact side. The expression in the lesioned side of the YL-YD group was significantly higher than that in the YL-OD and OL-OD groups. There was no significant difference between the YL-OD and OL-OD groups. There were significant differences for YL-YS versus YL-YD, YL-YS versus YL-OD, YL-YS versus OL-OD, OL-OS versus YL-YD, OL-OS versus YL-OD, and OL-OS versus OL-OD groups on the lesioned side (not indicated in the figure). **(C)** Preprodynorphin mRNA expression in the ventral striatum of lesioned side was increased in the YL-YD, YL-OD, and OL-OD groups compared with that on the intact side. The expression in the lesioned side of the YL-YD group was significantly higher than that of the OL-OD group. The expression levels in the YL-OD group were not significantly different from those in the YL-YD or OL-OD groups. There were significant differences for YL-YS versus YL-YD, YL-YS versus YL-OD, OL-OS versus YL-YD, OL-OS versus YL-OD, and OL-OS versus OL-OD groups on the lesioned side (not indicated in the figure). *p < 0.05; **p < 0.01. Error bars represent the standard error of the mean. L-dopa, levo-dihydroxyphenylalanine; OL-OD, old-lesioned old-L-dopa group; OL-OS, old-lesioned old-saline group; YL-OD, young-lesioned old-L-dopa group; YL-YD, young-lesioned young-L-dopa group; YL-YS, young-lesioned young-saline group.

Dopaminergic denervation alone at either young or old age did not affect striatal preprodynorphin mRNA expression (YL-YS and OL-OS groups in Figures 3A–C). Preprodynorphin mRNA expression in the dorsal striatum on the lesioned side was significantly increased compared to that in the intact side in all three groups with L-dopa treatment (YL-YD, YL-OD, and OL-OD groups). Further, mRNA expression in the dorsal striatum on the lesioned side of the YL-YD group was significantly higher than that in the YL-OD and OL-OD groups (Figures 3A,B) (groups, *F*[4,50] = 15.47, *p* < 0.001; sides, *F*[1,50] = 64.36, *p* < 0.001; groups^∗^sides, *F*[4,50] = 13.26, *p* < 0.001 by two-way repeated measures ANOVA) (*p* < 0.001 for the lesioned versus intact side in YL-YD, YL-OD, and OL-OD groups, *p* = 0.002 for YL-YD versus YL-OD group in the lesioned side, *p* < 0.001 for YL-YD versus OL-OD group in the lesioned side by Bonferroni *post hoc* test).

In the ventral striatum, preprodynorphin mRNA expression on the lesioned side was higher than that on the intact side in all three groups with L-dopa treatment (YL-YD, YL-OD, and OL-OD groups). mRNA expression in the ventral striatum on the lesioned side of the YL-YD group was significantly higher than that in the OL-OD group. There was no significant difference between YL-YD and YL-OD, or YL-OD and OL-OD groups (Figures 3A,C) (groups, *F*[4,50] = 11.35, *p* < 0.001; sides, *F*[1,50] = 28.93, *p* < 0.001; groups^∗^sides, *F*[4,50] = 6.60, *p* < 0.001 by two-way repeated measures ANOVA) (*p* < 0.001 for the lesioned versus intact side in YL-YD and YL-OD groups, *p* = 0.006 for the lesioned versus intact side in OL-OD group, *p* = 0.013 for YL-YD versus OL-OD group in the lesioned side, *p* = 0.863 for YL-YD versus YL-OD group in the lesioned side, *p* = 1.000 for YL-OD versus OL-OD group in the lesioned side by Bonferroni *post hoc* test).

Both denervation age and treatment age appeared to contribute to increased striatal preprodynorphin mRNA expression. The dorsal striatum is more crucially involved in motor control than the ventral (limbic) striatum (Haber, 2016). Accordingly, the expression difference in the dorsal striatum between the YL-YD and YL-OD groups is probably indicative of the more specific neurochemical hallmark of LID. Thus, these results suggest that treatment age is more important than denervation age, with the latter probably being involved in increased preprodynorphin mRNA expression.

**FIGURE 4:**
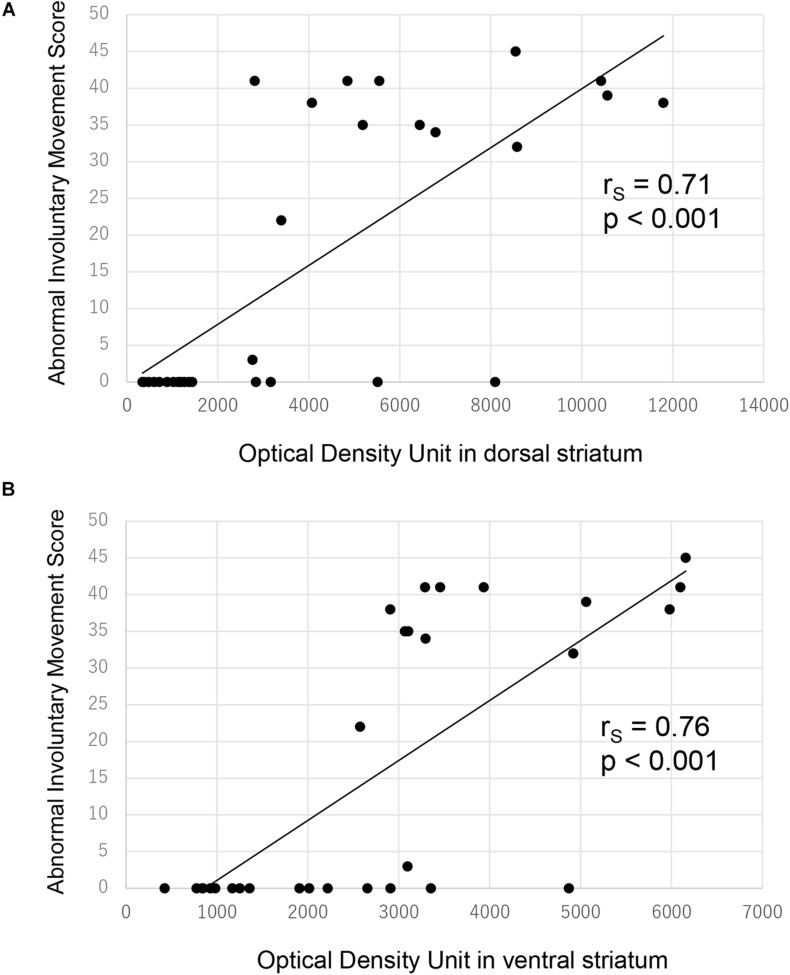
Correlation between ALO AIM score and preprodynorphin mRNA expression for all rats. There was significant correlation between ALO AIM score and preprodynorphin mRNA expression in the dorsal **(A)** and ventral **(B)** striatum. ALO AIM score, sum score of axial, forelimb, and orolingual scores; r_S_, Spearman’s rank correlation coefficient.

There were significant correlations between ALO AIM scores and preprodynorphin mRNA expression both in the dorsal (Figure 4A, *r*_S_ = 0.71, *p* < 0.001 by Spearman’s rank correlation coefficient) and ventral striatum (Figure 4B, *r*_S_ = 0.76, *p* < 0.001).

#### MGP Volume

**FIGURE 5:**
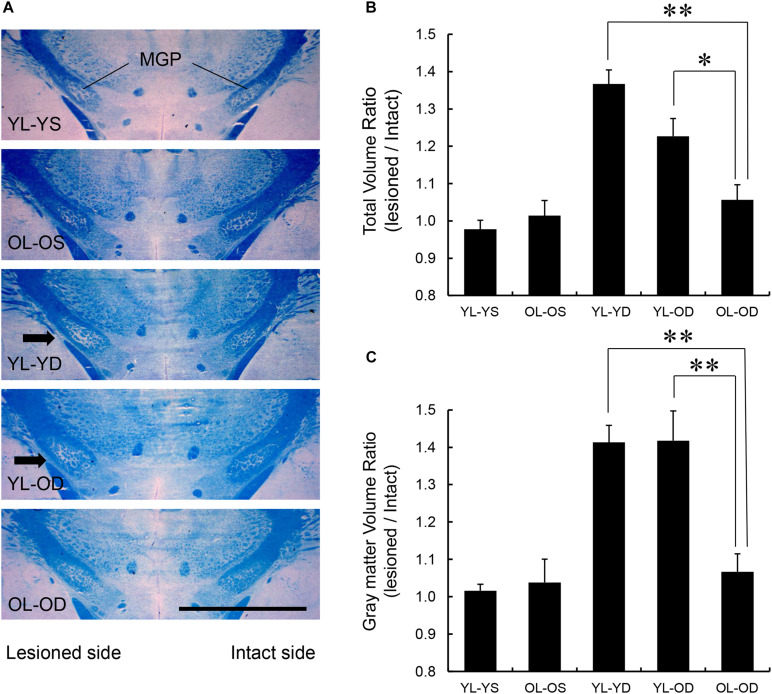
MGP volume in the five experimental groups. **(A)** Coronal frozen sections through the MGP of rats in each treatment group. Arrows in the images of YL-YD and YL-OD groups indicate MGP hypertrophy on the lesioned side. Klüver-Barrera stain. Scale bar, 3 mm. **(B)** Total volume ratio of the MGP of YL-YD group was significantly higher than that in the OL-OD group. The YL-OD group also showed increased ratio compared with that of the OL-OD group. There was no significant difference between the YL-YD and YL-OD groups. There were significant differences for YL-YS versus YL-YD, YL-YS versus YL-OD, OL-OS versus YL-YD, and OL-OS versus YL-OD groups (not indicated in the figure). **(C)** The gray matter volume ratio of the MGP of the YL-YD group was significantly higher than that of the OL-OD group. The YL-OD group showed increased ratio compared with that in the OL-OD group. There was no significant difference between the YL-YD and YL-OD groups. There were significant differences for YL-YS versus YL-YD, YL-YS versus YL-OD, OL-OS versus YL-YD, and OL-OS versus YL-OD groups (not indicated in the figure). *p < 0.05; **p < 0.01. Error bars represent standard error of the mean. L-dopa, levo-dihydroxyphenylalanine; MGP, medial globus pallidus; OL-OD, old-lesioned old-L-dopa group; OL-OS, old-lesioned old-saline group; YL-OD, young-lesioned old-L-dopa group; YL-YD, young-lesioned young-L-dopa group; YL-YS, young-lesioned young-saline group.

We found significant effects of groups on total MGP volume (*F*[4,25] = 17.82, *p* < 0.001 by one-way ANOVA). The volume ratios of the total MGP (lesioned to intact side) of the young-lesioned groups with L-dopa treatment (YL-YD and YL-OD groups) were significantly greater than those of the non-L-dopa-treated control groups (YL-YS or OL-OS groups) (*p* < 0.001 for YL-YD versus YL-YS group and YL-YD versus OL-OS group, *p* = 0.001 for YL-OD versus YL-YS group, *p* = 0.006 for YL-OD versus OL-OS group by Tukey–Kramer *post hoc* test) (Figures 5A,B). The ratio of the OL-OD group was not significantly different from that of controls (*p* = 0.612 for OL-OD versus YL-YS group, *p* = 0.938 for OL-OD versus OL-OS group). The YL-YD and YL-OD groups showed significantly increased ratios compared to the OL-OD group (*p* < 0.001 for YL-YD versus OL-OD group, *p* = 0.034 for YL-OD versus OL-OD group) (Figure 5B). There was no significant difference between the YL-YD and YL-OD groups (*p* = 0.113) (Figure 5B). These results indicate that denervation age is important for the MGP enlargement, a pathological hallmark of LID (Tomiyama et al., 2004; Nishijima et al., 2020). Specifically, the difference between the YL-OD and OL-OD groups suggests that denervation at a young age independently contributes to this enlargement.

We found significant effects of groups on gray matter MGP volume (*F*[4,25] = 14.21, *p* < 0.001 by one-way ANOVA). The volume ratios of the gray matter of the MGP were increased in the YL-YD and YL-OD groups compared with those in the YL-YS, OL-OS, and OL-OD groups (*p* < 0.001 for YL-YD versus YL-YS group, YL-YD versus OL-OS group, YL-OD versus YL-YS group and YL-OD versus OL-OS group, *p* = 0.001 for YL-YD versus OL-OD group, *p* = 0.001 for YL-OD versus OL-OD group by Tukey–Kramer *post hoc* test) (Figure 5C). There was no significant difference between the YL-YD and YL-OD groups (*p* = 1.000) (Figure 5C). The difference between the YL-OD and OL-OD groups implies that dopaminergic denervation at a young age is important for gray matter enlargement in the MGP. Increased MGP volume mainly results from enlargement of axon terminals of striatum-MGP projection neurons, the direct pathway neurons (Nishijima et al., 2020). Accordingly, the increase in gray matter volume (Figure 5C) is a more specific pathological hallmark of LID. Thus, denervation age, not treatment age, enhanced MGP hypertrophy with L-dopa treatment in PD rats.

### Tyrosine Hydroxylase Immunohistochemistry

**FIGURE 6:**
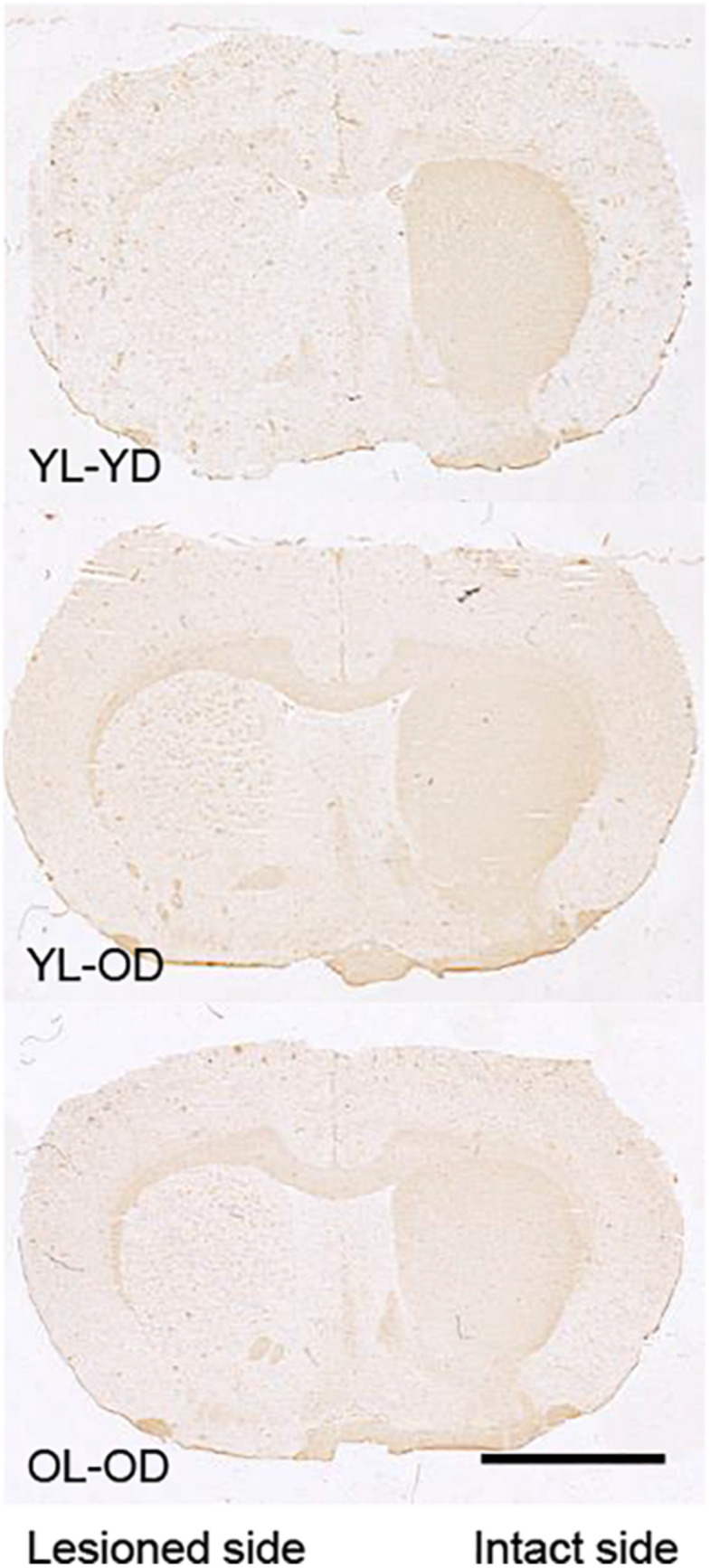
Tyrosine hydroxylase immunohistochemistry in the striatal sections of 6-OHDA-lesioned and L-dopa-treated rats. The lesioned striatal side did not stain for tyrosine hydroxylase in any group. Scale bar, 3 mm. 6-OHDA, 6-hydroxydopamine; L-dopa, levo-dihydroxyphenylalanine; OL-OD, old-lesioned old-L-dopa group; YL-OD, young-lesioned old-L-dopa group; YL-YD, young-lesioned young-L-dopa group.

The lesioned side of the striatum did not stain for tyrosine hydroxylase in the YL-YD, YL-OD, and OL-OD groups (Figure 6), indicating that almost complete dopaminergic denervation was achieved with our experimental procedures.

## Discussion

This study shows that young-lesioned PD model rats have an increased propensity for developing LID-like movements than do old-lesioned PD rats, as reported in patients with young-onset PD (Schrag and Quinn, 2000; Kumar et al., 2005; Ku and Glass, 2010; Olanow et al., 2013). Dopaminergic denervation at a young age appeared to be crucially involved in the propensity among PD models with MGP hypertrophy. However, L-dopa treatment at a young age also seemed to be involved in susceptibility to LID in young-lesioned PD model rats, as evidenced by increased striatal preprodynorphin mRNA expression.

Two previous studies on LID rodent models with 6-OHDA-induced dopaminergic denervation at an old age (Bez et al., 2016; Lanza et al., 2019) showed that old-age dopaminergic denervation enhanced L-dopa-induced abnormal movements compared with denervation at a young age, which is inconsistent with our results and clinical observations. To develop a PD model, Bez et al. (2016) used female C57Bl/6J mice and injected 6-OHDA into the striatum (with 50–70% dopamine neuron loss); in contrast, we injected 6-OHDA into the medial forebrain bundle (with 90–100% dopamine neuron loss) (Blandini and Armentero, 2012). Additionally, we performed a confirmation test for dopaminergic denervation using apomorphine before behavioral analyses. Lanza et al. (2019) used male Fischer 344 rats and injected 6-OHDA into the medial forebrain bundle, a method similar to ours, to induce dopaminergic denervation but did not confirm the degree of dopaminergic denervation before L-dopa treatment. In our confirmation test in the present study, 75% of the young-lesioned rats met our criteria for almost complete dopaminergic denervation. We excluded rats that did not meet the criteria from the present study because the behavioral response to L-dopa in PD animal models is very sensitively affected by the degree of dopaminergic denervation. The inconsistency in findings can be attributed to varying degrees of nigrostriatal dopaminergic denervation. Furthermore, dopamine neurons of old rats are more vulnerable to 6-OHDA than those of young rats (Barata-Antunes et al., 2020). Accordingly, elderly lesioned animals could have more severe dopamine neuron loss than young-lesioned rats before the behavioral analyses, which could explain the increasingly severe dyskinetic movements (Bez et al., 2016; Lanza et al., 2019), although the striatal dopamine contents at the end of the experiment by Lanza et al. (2019) were not so different between young- and old-lesioned model rats. Notably, the apomorphine challenge has a priming effect on motor sensitization in 6-OHDA-treated rats, which should also be taken into consideration (Henry et al., 1998). Moreover, differences in animal strains, sex, and L-dopa dose and behavioral testing schedule (after single L-dopa exposure or chronic repetitive treatment) should have an impact on the controversial results.

Our findings that the young-lesioned PD model was vulnerable to LID-like movements is consistent with clinical observations that young-onset patients with PD are more likely to develop LID (Schrag and Quinn, 2000; Kumar et al., 2005; Ku and Glass, 2010; Manson et al., 2012; Olanow et al., 2013). Additionally, the present observation that young-lesioned PD models are susceptible to L-dopa-induced LID-like movements both with early and late L-dopa treatment, is consistent with a clinical report that disease severity, but not L-dopa treatment duration, was associated with LID expression in the Ghanaian and Italian cohorts (Cilia et al., 2014). This suggests that dopaminergic denervation, but not treatment, at a young age plays a main role in LID propensity among young-lesioned PD models. However, analyses of L-dopa-induced rotational behavior and a biochemical marker, preprodynorphin, indicated that L-dopa treatment at a young age may also be associated with propensity for LID.

Sensitization of rotational behavior to repetitive L-dopa treatment is considered to have common mechanisms as LID development (Henry et al., 1998). In the present study, there was no significant different in L-dopa-induced rotational behavior between three L-dopa treated groups at the first treatment day, suggesting the severity of 6-OHDA lesion is at the same level between the groups. After 14-day chronic repetitive treatment with L-dopa, both the dopaminergic denervation and L-dopa treatment at a young age contributed to a marked sensitization in PD models, indicating that the age of the 6-OHDA lesion and L-dopa treatment, not the severity of the 6-OHDA lesion, contributes to the priming for enhanced rotational behavior. L-dopa treatment at a young age is more involved in increased preprodynorphin mRNA expression in the dorsal “motor” striatum, a neurochemical hallmark of LID. The increased MGP gray matter, a pathological hallmark of LID, in both YL-YD and YL-OD groups implies that denervation at a young age contributes to the MGP enlargement. Taken together, both dopaminergic denervation and L-dopa treatment at a young age contribute to susceptibility for LID in young-lesioned PD models.

The mechanisms underlying the enhanced susceptibility to LID in young-onset PD remain unclear. Generally, neuronal plasticity declines with age (Bergado and Almaguer, 2002; Yankner et al., 2008). Since LID results due to abnormal plasticity, young rats could have a higher propensity for maladaptive changes than old rats. Dopaminergic denervation induces plastic changes in brain motor circuits, such as spine loss on dendrites of the striatal projection neurons (Day et al., 2006; Villalba et al., 2009; Fieblinger et al., 2014; Nishijima et al., 2014; Suarez et al., 2014, 2016, 2018; Toy et al., 2014; Gagnon et al., 2017; Gomez et al., 2019; Graves and Surmeier, 2019). It rapidly induces serotonergic hyperinnervation in the striatum (Maeda et al., 2003), which may lead to excessive dopamine release into the striatum after L-dopa treatment (Tanaka et al., 1999). Moreover, dopamine loss alters the expression and sensitivity of the somatic D1 and D2 dopamine receptors in the striatum (Hornykiewicz, 2001; Gerfen, 2003; Corvol et al., 2004; Aubert et al., 2005; Guigoni et al., 2007). Presynaptic D1 receptors on striatonigral axon terminals also become supersensitive after dopamine neuron loss in the substantia nigra (Ding et al., 2015). Additionally, dopaminergic denervation induces the loss of negative feedback via GABA_B_ receptor in the axon terminal of striatal spiny projection neurons of the direct pathway, leading to neuronal hyperactivity and LID (Borgkvist et al., 2015). These plastic changes with dopaminergic denervation in the brain might be more extensive in young animals, which remains to be investigated. Further, dopamine-denervation-induced plasticity results in marked fluctuation of dopamine release in the striatum after L-dopa treatment, which could be more severe in young PD models. We found that L-dopa-induced MGP hypertrophy was more evident in the young-lesioned PD model. We recently found that MGP hypertrophy is a hallmark of LID in the rat model of PD. Hypertrophy results due to the enlargement of axon terminals of the spiny projection neurons in the direct pathway projecting from the striatum to MGP. The enlargement was characterized by excessive GABA storage packed in synaptic vesicles (Nishijima et al., 2020). Severe MGP hypertrophy in young-lesioned PD model rats might result due to a marked swing in dopamine release into the striatum after L-dopa treatment (Tanaka et al., 1999). Both denervation- and L-dopa-induced plasticity are present in PD models with repeated L-dopa treatment. Accordingly, it seems reasonable that both denervation and treatment age contribute to the development of LID-like movements. Our model rats would be useful for further studies to examine these presumed age-related mechanisms.

There are several limitations to the present study. First, we did not perform quantitative analyses of the dopamine content in the denervated striatum of our model rats; however, we have previously confirmed that our procedure produces almost complete hemi-dopaminergic denervation (Tanaka et al., 1999). Second, we used toxin-induced hemi-parkinsonian rats. Our results using an acute dopaminergic denervation model may not be necessarily translated to aging effects on neurodegenerative processes in the patients. Third, we did not examine the effect of aging on striatal serotonergic hyperinnervation or striatal neuronal hyperactivity after dopaminergic denervation. Forth, biochemical parameters other than preprodynorphin mRNA expression have not been analyzed in the present study, which should be examined in future studies.

In conclusion, we demonstrated, to the best of our knowledge, for the first time that the dopaminergic denervation at a young age contributes to the development of severe LID in PD model rats. The initiation of L-dopa at a young age also partly contributes to LID development. Future studies on the effect of aging on plastic changes in brain circuits including the basal ganglia after dopaminergic denervation should be performed. Studies using genetic models of PD are also warranted.

## Data Availability Statement

The raw data supporting the conclusions of this article will be made available by the authors, without undue reservation.

## Ethics Statement

The animal study was reviewed and approved by Hirosaki University School of Medicine.

## Author Contributions

HN: contributed to the conceptualization, data curation, formal analysis, investigation, visualization, and writing – original draft preparation. TaK and FM: contributed to the investigation, methodology, visualization, writing – review and editing. KW: contributed to the methodology, resources, and writing – review and editing. IK, TN, ToK, and CS: contributed to the investigation, and writing – review and editing. MT contributed to the conceptualization, data curation, formal analysis, funding acquisition, investigation, methodology, project administration, resources, supervision, and writing – review and editing. All authors contributed to the article and approved the submitted version.

## Conflict of Interest

The authors declare that the research was conducted in the absence of any commercial or financial relationships that could be construed as a potential conflict of interest.

## Supplementary Material

Supplementary Video 1 Mild limb dyskinesia induced by L-dopa treatment for a right sided 6-OHDA-lesioned rat. We evaluated this level of dyskinesia with a score of 2 on the severity scale.

Supplementary Video 2 Severe limb dyskinesia induced by L-dopa treatment for a right sided 6-OHDA-lesioned rat. We evaluated this level of dyskinesia with a score of 4 on the severity scale.

Supplementary Video 3 Mild to moderate rotational behavior induced by L-dopa treatment for a right sided 6-OHDA-lesioned rat.

Supplementary Video 4 Severe rotational behavior induced by L-dopa treatment for a right sided 6-OHDA-lesioned rat. 6-OHDA, 6-hydroxydopamine; L-dopa, levo-dihydroxyphenylalanine.

## Abbreviations

6-OHDA: 6-hydroxydopamine
ABC: avidin-biotin-peroxidase complex
AIM: abnormal involuntary movement
ALO: axial forelimb and orolingual
ANOVA: analysis of variance
APS: aminopropyltriethoxysilane
GABA: gamma-aminobutyric acid
L-dopa: levo-dihydroxyphenylalanine
LID: L-dopa-induced dyskinesia
MGP: medial globus pallidus
NIH: National Institutes of Health
OL: old-lesioned
OL-OD: old-lesioned old-L-dopa
OL-OS: old-lesioned old-saline
PD: Parkinson’s disease
mRNA: messenger ribonucleic acid
YL: young-lesioned
YL-OD: young-lesioned old-L-dopa
YL-YD: young-lesioned young-L-dopa
YL-YS: young-lesioned young-saline.
